# *flam* piRNA precursors channel from the nucleus to the cytoplasm in a temporally regulated manner along *Drosophila* oogenesis

**DOI:** 10.1186/s13100-019-0170-7

**Published:** 2019-07-06

**Authors:** Cynthia Dennis, Emilie Brasset, Chantal Vaury

**Affiliations:** GReD, Université Clermont Auvergne, CNRS, INSERM, Faculté de Médecine, 63000 Clermont-Ferrand, France

**Keywords:** Transposable elements, Silencing, piRNAs, *Flamenco*, Dot COM, Yb-body, Drosophila, Oogenesis

## Abstract

**Background:**

PIWI-interacting RNAs (piRNAs) are the effectors of transposable element silencing in the reproductive apparatus. In *Drosophila* ovarian somatic cells, piRNAs arise from long RNA precursors presumably processed within cytoplasmic Yb-bodies.

**Results:**

Here we show that the nucleo-cytoplasmic traffic of piRNA precursors encoded by the *flamenco* locus is subjected to a spatio-temporal regulation. Precursor RNAs first gather in a single nuclear focus, Dot COM, close to the nuclear periphery, and transit through the membrane before being delivered to the cytoplasmic Yb-bodies. Early in oogenesis, *flamenco* transcripts are rapidly transferred to the cytoplasm making their initial nuclear gathering in Dot COM too transient to be visualized. As oogenesis proceeds, the cytoplasmic delivery steadily decreases concomitantly with the decrease in the protein levels of Armi and Yb, two components of the Yb-bodies. Both events lead to a reduction of Yb-body assembly in late stages of oogenesis, which likely results in a drop in piRNA production.

**Conclusion:**

Our findings show a spatio-temporal regulation of the piRNA biogenesis in the follicle cells of *Drosophila* ovaries, that involves coordinated control of both piRNA precursors and components of the piRNA processing machinery. This newly unveiled regulation establishes another level of complexity in the production of piRNAs and suggests a stage-dependent involvement of the piRNA biogenesis in the mechanism of transposable elements silencing along oogenesis.

**Electronic supplementary material:**

The online version of this article (10.1186/s13100-019-0170-7) contains supplementary material, which is available to authorized users.

## Background

Eukaryotic genomes are composed of a variable proportion of transposable elements (TEs) accumulated throughout evolution. These sequences are silenced by the host to protect itself and its progeny against potentially deleterious mutations and genome invasion. In the gonads, where it is essential to ensure the maintenance of genome integrity for the next generation, the piRNA pathway is responsible for TE silencing in both somatic and germinal tissues [1–4]. This process involves small guide piRNAs of 23–29 nucleotides (nts) that originate from discrete genomic regions called piRNA clusters.

142 piRNA clusters have been identified in *Drosophila melanogaster* [2], mostly located in pericentromeric and telomeric regions. These clusters vary considerably in size from a few kilobases (kb) to several hundred kb. They are enriched in full length or truncated TEs that are often nested within one another [2, 5]. In ovarian somatic support cells surrounding the germline, piRNAs are mainly produced from two piRNA clusters: *traffic jam* [6] and *flamenco* (*flam*) [2, 7]. The *flam* cluster is located at the pericentromeric region of the X-chromosome, spans over more than 200 kb and is strongly enriched in both ancient and recent retrotransposons mostly inserted in an orientation antisense to the TE transcription [5]. *flam* is transcribed from a polymerase II promoter as a long single-stranded RNA that is a substrate for piRNA biogenesis. *Flam* transcripts undergo alternative splicing to generate diverse piRNA precursors that all share the first exon at their 5′ end [8]. These transcripts are then processed into 23–29 nt piRNAs, presumably in cytoplasmic Yb-bodies [9, 10]. Mature piRNAs associated with Piwi protein form a piRNA-induced silencing complex (piRISC) that is delivered to the nucleus to target nascent TE mRNAs and initiate transcriptional gene silencing [11, 12].

Two studies have reported that *flam* precursor transcripts, together with transcripts from other piRNA clusters, concentrate in 1 to 2 foci in ovarian follicle cells [13, 14]. Analysis of the subcellular localization of these sites of accumulation at different stages of development have yielded varying findings. The first study focused on follicle cells in late stages of oogenesis and showed that *flam* RNA precursors accumulate in a single nuclear substructure, named Dot COM, that faces the cytoplasmic Yb-body to which the *flam* precursors are channeled by nucleo-cytoplasmic transfer [13]. A subsequent report [14] visualized *flam* transcripts concentrated within the cytoplasm in 1 to 2 substructures named *flam* bodies, located close to Yb-bodies. The authors worked mainly with a cultured *Drosophila* ovarian somatic stem cell line (OSS cells) derived from a somatic stem cell population of the germarium [15] that expresses a functional piRNA pathway [6].

We show here that *flam* transcripts are channeled to the cytoplasm in a temporally regulated manner. In early stages of oogenesis, they are mainly detected within the cytoplasm. As oogenesis proceeds, *flam* transcripts accumulate in a focus detected within the nucleus or within the nuclear membrane as though their delivery to the cytoplasm was impeded. Combined with a drop in Armi and Yb protein levels after stage 8, this affects the assembly of Yb-bodies which then are very small and absent from most cells. These findings emphasize the temporal regulation of Yb-body assembly, which requires both cytoplasmic *flam* delivery and Armi and Yb proteins. In their absence, Yb-bodies fail to assemble correctly, which potentially can cause a decrease in the production of piRNAs.

## Results and discussion

### The cytoplasmic transfer of *flam* precursor transcripts decreases during ovariole development

In a previous study [16], we performed a global quantitative analysis of the subcellular localization of *flam* transcripts within ovarian somatic follicle cells, independently of the developmental stages of oogenesis. Depending on the follicle cell observed, the focus where *flam* precursors gathered was visualized either completely within the nucleus close to the nuclear periphery, or stretching across the nuclear membrane, or within the cytoplasm close to the nuclear membrane. To investigate whether this differential localization is somehow related to the stage of the developing egg chambers, we examined *flam* RNA precursors in follicle cells within egg chambers in stages 3 to 10 of oogenesis. To do so, we performed an immuno-RNA FISH experiment on WT *Drosophila* ovaries using an anti-lamin antibody to label the nuclear membrane, and a specific *flam* RNA probe described in [13]. The same experiment was performed in parallel with OSS cells. The localization of *flam* foci was quantified as follows. When positioned close to the inner side or outer side of the lamin signal, foci were considered as nuclear within Dot COM or cytoplasmic within the Yb-bodies respectively. When co-localized with the lamin signal (totally or partially), the *flam* focus was considered as stretching across the nuclear membrane.

**Fig. 1:**
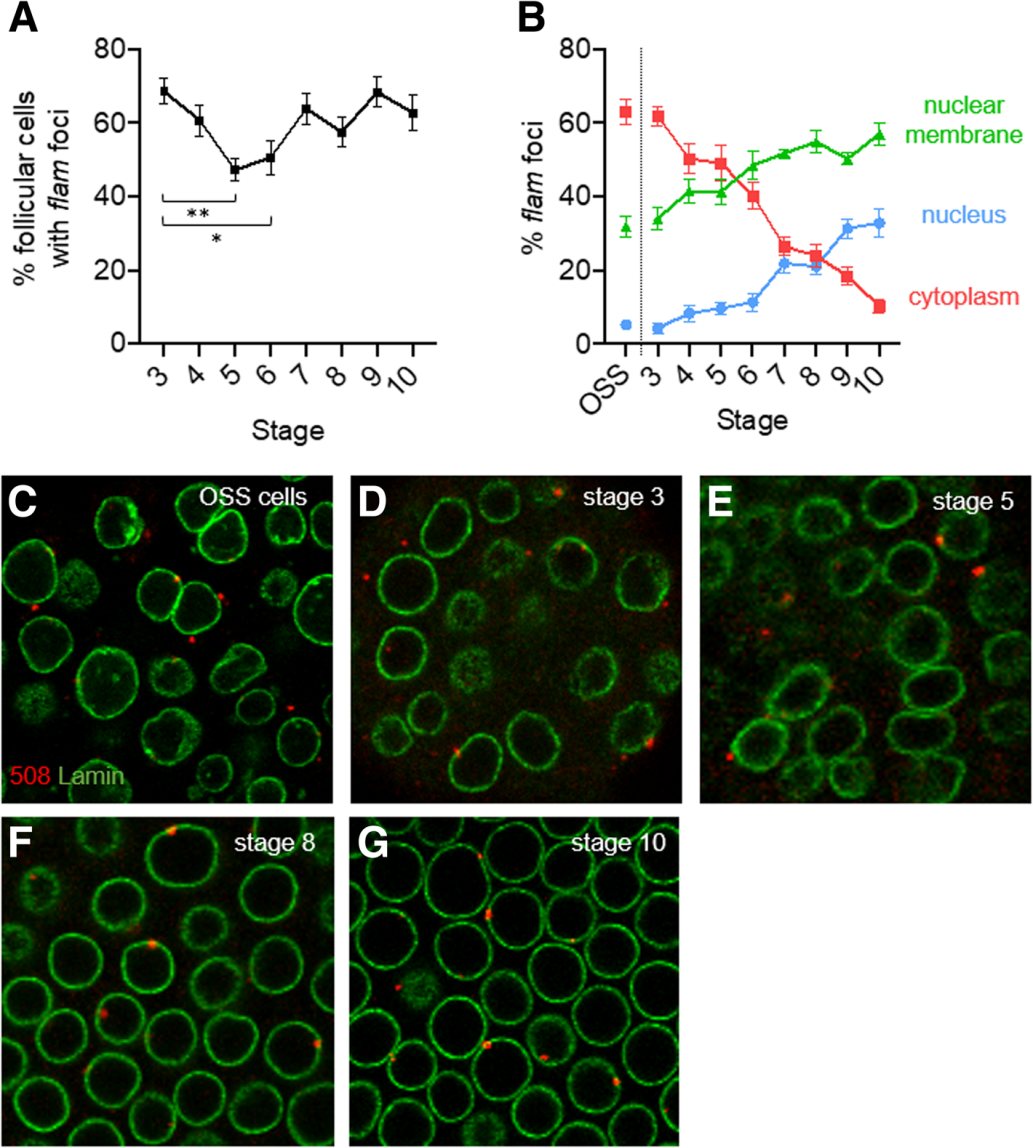
Sub-cellular localization of *flam* focus along oogenesis and in OSS cells. **a** Quantitative analysis of the percentage of follicles cells from eggs chambers of stage 3 to 10 with detectable *flam* foci. **b** Quantitative analysis of *flam* foci localization in OSS cells and in follicle cells from stages 3–10 of oogenesis. The percentage of *flam* foci localized in the cytoplasm (red), in the nucleus (blue) and at the nuclear membrane (green) for OSS cells and each stage of development is plotted. Error bars represent s.e.m. Numbers of cells and follicles counted is indicated in Additional file 3: Table S1. **c**-**g** Subcellular localization of *flam* foci in OSS cells (**c**) or ovarian follicle cells of stage 3 (**d**), stage 5 (**e**), stage 8 (**f**) or stage 10 (**g**). *flam* transcripts visualized as 1 to 2 foci per cell are labelled in red using *flam* 508 RNA probe and nuclear membrane is labelled in green with anti-lamin antibody

Firstly we investigated egg chambers from stage 3 to 10 of oogenesis and quantified the proportion of cells in which a *flam* signal could be visualized (Fig. 1a). We found no significant difference between stages 3, 4 and 7–10 where *flam* foci are detected in more than 60% of cells. This proportion appears slightly lower around 50% at stages 5 and 6 (Fig. 1a).

Secondly, focusing on the follicle cells that harbored a *flam* signal, we investigated its subcellular localization. We found that *flam* transcripts that accumulated mostly in a single focus, vary drastically in localization from early to late stages of oogenesis. In early stages, in a majority of follicle cells, 1 to 2 *flam* foci are observed within the cytoplasm (Fig. 1b, d, e). OSS cells, which derive from a somatic stem cell population of the germarium, and follicle cells from early stage 3, display a similar percentage of *flam* foci within the cytoplasm (around 62%) (Fig. 1b & c). This similarity may be explained by the intrinsic nature of these two cell types; both are highly dividing cells required to rapidly encapsulate the 16-cell germinal cyst. The percentage of cells with cytoplasmic *flam* foci decreases progressively to 10% at stage 10 of oogenesis. Concomitantly, the proportion of follicle cells in which a *flam* focus is detected within Dot COM increases, from 4% in stage 3 to 32% in stage 10 (Fig. 1b-g). The switch from a cytoplasmic to a nuclear position is progressive, suggesting that the export of piRNA precursors to the cytoplasm steadily decreases as oogenesis proceeds. This stage-by-stage analysis further emphasizes that an increasing fraction of *flam* RNAs co-localizes with the lamin staining. Interestingly, in late stages of oogenesis, the *flam* transcripts are even more likely to be detected spanning the nuclear membrane (57%) than within the nucleus (33%) and the cytoplasm (10%) (Fig. 1b). These data indicate that *flam* precursors can enter the nuclear membrane throughout oogenesis even in late stages but seem to lose progressively the ability to be delivered to the cytoplasm. This suggests that fewer piRNA precursors reach the Yb-bodies, thereby giving rise to fewer piRNAs in late stages of oogenenesis.

### At early stages of Drosophila oogenesis, *flam* precursors rapidly transit to the cytoplasm

**Fig. 2:**
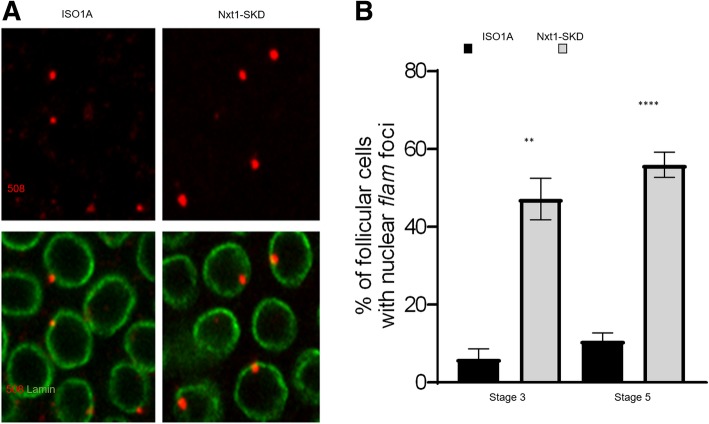
At early stages, *flam* precursors can be visualized accumulated in the nucleus**. a** Sub-cellular localization of *flam* transcripts in WT ISO1A or *Nxt1*-SKD follicle cells of stage 5. *flam* transcripts are labelled in red using *flam* 508 RNA probe and nuclear membrane is labelled in green with anti-lamin antibody. **b** Quantitative analysis of *flam* nuclear foci localization in WT ISO1A or *Nxt1*-SKD follicle cells, at stage 3 and 5 of oogenesis. Error bars represent s.e.m. Numbers of cells and follicles counted is indicated in Additional file 3: Table S1

To better understand the discrepancy between *flam* localization in early and late stages of oogenesis, we first considered the early stages of oogenesis and posed the question of why no nuclear accumulation is observed. The nuclear gathering in Dot COM could be specific to later stages of oogenesis and not occur in early stages. Alternatively, the nuclear accumulation could be too transient before export to be detected in our immuno-RNA FISH experiments. To distinguish between these two hypotheses, we examined the localization of *flam* transcripts in follicle cells of *Nxt1-*SKD flies in early stages of oogenesis (*Nxt1* somatic knockdown using *tj-Gal4* driver) when the export of *flam* precursors is impeded. Nxt1 protein is a RNA export factor that is required, together with its partner Nxf1, for the export of *flam* precursors [16]. We observed a clear increase in the proportion of follicle cells with a nuclear accumulation of *flam* transcripts in early stages 3 and 5 in *Nxt1-*SKD compared to WT ISO1A (Fig. 2 a & b), which suggests that nuclear accumulation of *flam* precursors can occur in early stages of oogenesis.

These findings indicate that, at early stages, in their traffic to the cytoplasm, *flam* transcripts are shuttled to the cytoplasm very rapidly. Export of the piRNA precursors is thus more efficient in early than in late stages of oogenesis.

### At late stages of Drosophila oogenesis, *flam* precursors fail to be delivered to the cytoplasm

The second issue we addressed was to understand why the delivery of *flam* precursors from the membrane to the cytoplasm is steadily affected as oogenesis proceeds. We have previously shown that the export of *flam* precursor transcripts to the cytoplasm is the signal for the Yb-bodies to be assembled [5]. If, as suggested above, *flam* precursors do not reach the cytoplasm in late stages, then the proportion of Yb-bodies is expected to decrease.

**Fig. 3:**
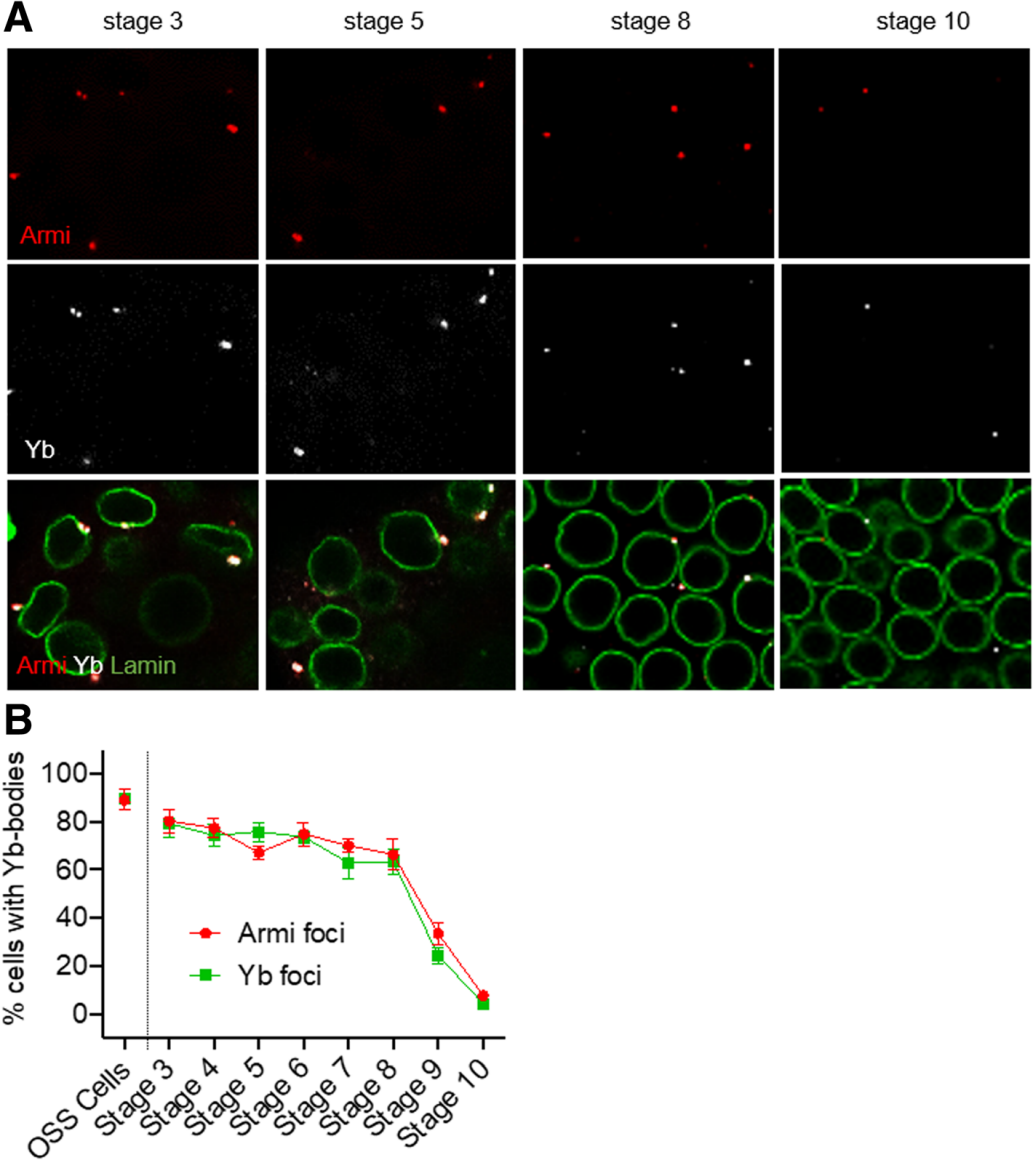
Armi and Yb protein levels are temporally regulated in follicle cells along oogenesis. **a** Armi (red) and Yb-foci (white) and nuclear membrane (green) are visualized by immunofluorescence using respectively anti-Armi, anti-Yb and anti-lamin antibodies in ovarian follicle cells at stages 3, 5, 8 and- 10 of oogenesis. **b** Quantitative analysis of the percentage of cells exhibiting at least one Armi-focus (red) or one Yb-body (green) in OSS cells and at stages 3–10 of oogenesis. The percentage of cells bearing one or more foci at each indicated stage of development is plotted. Error bars represent s.e.m. Numbers of cells and follicles counted is indicated in Additional file 3: Table S1

In immunostaining experiments using Armi and Yb antibodies, we indeed observed that the size of the Yb-bodies (Fig. 3a) detected in follicle cells decreases during oogenesis together with the percentage of cells that have a discernable Yb-body (Fig. 3b). At stage 10 less than 10% of cells have Armi and Yb foci compared to 80% in stage 3 or 90% in OSS cells (Fig. 3b and Additional file 1: Figure S1A).

However, the decrease in Yb-body assembly in late stages of oogenesis cannot be attributed solely to the reduction of *flam* piRNA precursors exported to the cytoplasm. In the germarium, the follicle cells encapsulate the germline cyst and carry out a mitotic division program from stage 2 to stage 6 of oogenesis [17]. At stage 6, they cease to undergo mitotic cycles and start endocycles. This transition is regulated by the Notch pathway [17]. It has been demonstrated that Armi protein level decreases at this switch from mitosis to endoreplication owing to Notch signaling pathway activation [18]. Immuno-histochemical experiments performed with Armi and Yb- antibodies on whole ovaries showed that not only is Armi protein level reduced in late stages of oogenesis, but also Yb staining. We observed a clear drop during oogenesis with a high level of Armi and Yb proteins only until stage 8 and a weak staining afterwards (Fig. 3b & Additional file 1: Figure S1) indicating that both proteins are presumably regulated at the transition from mitosis to endoreplication.

**Fig. 4:**
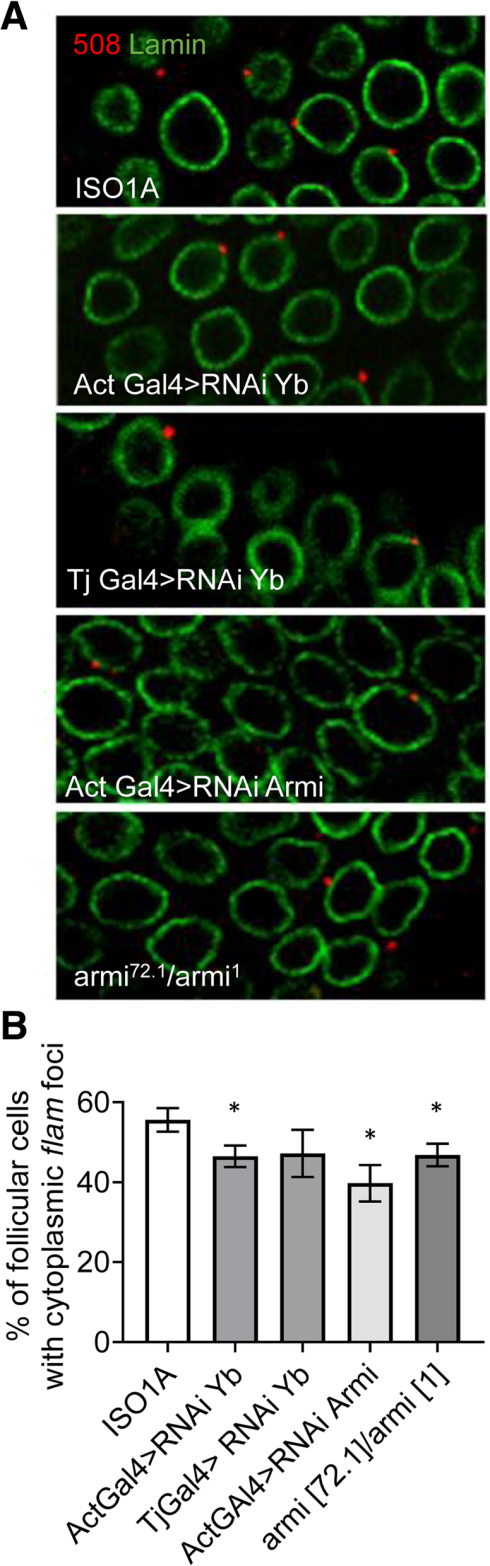
*flam* piRNA precursor export in early stages is slightly impeded in armi- and yb-depleted cells. **a** *flam* transcripts (red) and nuclear membrane (green) in stages 5 of oogenesis are visualized by RNA-FISH and immunofluorescence using respectively *flam* 508 RNA probe and anti-lamin antibody in follicle cells of *ISO1A* line, RNAi knockdown of *Yb* and *armi* and trans-heterozygous for *armi*. **b** Quantitative analysis of cytoplasmic *flam* foci localization at stage 5 of oogenesis in the *Drosophila* lines indicated. Error bars represent s.e.m. Numbers of cells and follicles counted is indicated in Additional file 3: Table S1

To determine whether the reduced export of *flam* transcripts at late stage of oogenesis is related to the decrease in Armi and Yb protein levels after stage 8, we analyzed whether a reduction of Armi or Yb protein level in early stages of oogenesis would have the same impact on RNA precursor export. To do so we quantified the sub-cellular localization of *flam* transcripts in follicle cells of stage 5 depleted for Armi or Yb using RNAi or mutant lines. The localization of *flam* transcripts was found similar between WT and *Armi*- and *Yb*-depleted lines, with a majority of follicle cells having transcripts located within the cytoplasm. However, quantifications showed a low but significant decrease in the proportion of cells exhibiting cytoplasmic *flam* foci (Fig. 4a & b). A reduced accumulation of *flam* transcripts in the cytoplasm may result from an increased instability of the transcripts due to the absence of Armi and Yb in the cytoplasm. Alternatively, the mild decrease in the proportion of cells with cytoplasmic *flam* foci is expected if the export of *flam* transcripts is impeded. It has been suggested that *armi* is expressed in the follicle cells of the trans-heterozygous mutant *armi*^*1*^*/armi*^*72.1*^ [18] and we cannot exclude an incomplete depletion of Armi and Yb proteins in early stages in the RNAi lines used. Nevertheless, these mutants reveal a possible involvement of Armi and Yb proteins in the delivery of piRNA precursors to the cytoplasm. It has previously been shown that Armi and Yb proteins bind piRNA precursors [19–22]. It has also been reported that artificially tethering Armi or Yb protein to a heterologous RNA channels this RNA to the cytoplasmic processing machinery [23, 24]. Armi ATP-dependent helicase activity has also been observed in vitro [24] and been implicated in Armi specific binding to piRNAs precursors and piRNA production [21, 22, 25], with the hypothesis that Armi binds and unpacks piRNA precursors while being exported to the cytoplasm. Whether this binding happens in the nucleus or at the nuclear membrane remains to be elucidated. In late stages of oogenesis in *Nxt1*-SKD flies, we observed that Armi but not Yb protein can occasionally be visualized within the nucleus as a dotty pattern (Additional file 2: Figure S2). Of the 3074 follicle cells examined from 20 stage 10 egg chambers, Armi was detected in 7.9% of the nuclei, along with a great variability among the follicles (0 to 18%). This raises the possibility that Armi protein shuttles between the cytoplasm and nucleus which is not detectable in WT conditions. However, we cannot exclude that this Armi pattern of staining is a consequence of a detrimental lack of the Nxt1 export protein in the ovarian follicle cells.

On the basis of these findings, we propose that two interconnected events occur during oogenesis that lead to a drop in Yb-body assembly in late stages. On the one hand, following Notch-signaling pathway activation in stage 6–7, the levels of Armi and Yb proteins are drastically reduced, which not only decreases the assembly of the Yb-bodies but also impedes the export of piRNA precursors (Fig. 4). On the other hand, the export of piRNA precursors is progressively reduced along oogenesis which further hampers the assembly of the remaining Armi and Yb into Yb-bodies.

Reduction of the assembly of Yb-bodies and of the export of *flam* piRNA precursors is thought to cause a decrease in the production of piRNAs in late stages of oogenesis. However, several studies indicate that the piRNA silencing is active throughout oogenesis. For example, if we consider the two retrotransposons, ZAM and gypsy, their enhancers are active at the posterior pole of the follicle cells throughout oogenesis including in late stages [26, 27]. Nevertheless, their transcripts are never detected in WT conditions owing to the silencing exerted by the piRNA pathway, which indicates that the silencing exerted on ZAM and gypsy is active even in late stages. One possible explanation of TE silencing in late stages of oogenesis when piRNA production seems impeded could be that the few and highly diminished Yb-bodies present in late stages give rise to a piRNA population that is sufficient to silence TEs. Alternatively, and not exclusively, piRNAs produced anteriorly in early stages are stable enough to remain active in late stages. Finally, it cannot be excluded that a transcriptional silencing exerted in early stages can still prevent transcription of the TEs in late stages with no need for piRNAs. The differential quantification of mature piRNAs produced in early versus late stages of oogenesis will provide a far better view of piRNAs produced during oogenesis.

## Conclusions

From transcription to processing, multiple steps and numerous protein factors are required to drive piRNA precursors into the cytoplasmic structure where functional piRNAs are produced. In the follicle cells of *Drosophila* ovaries, RNAs transcribed from piRNA clusters are first spliced, presumably concomitantly to transcription [8], before being transferred throughout the nucleoplasm in an Exon Junction complex, UAP56 protein and Nxt1-Nxf1-dependent manner, to be assembled in a single nuclear focus [13, 16]. Then, the transfer to the cytoplasm requires the export complex Nxt1-Nxf1 and our present study suggests that Armi and Yb proteins may somehow be implicated in this process (Fig. 4). Armi protein is thought to be able to bind several types of RNAs being exported - including mRNAs. In the cytoplasm, its specific interaction with piRNA precursors is regulated by its ATPase hydrolysis activity and Yb protein [21, 25]. Our present study suggests that Armi could play an earlier role within the nucleus before RNA export. When in the cytoplasm, piRNA precursors are delivered to the Yb-bodies and mitochondria where Yb and Armi play distinct and collaborative roles to ensure the production of Zuc-dependent phased piRNAs [21, 25].

Our study shows that a spatially and temporally controlled piRNA biogenesis exists with two critical periods taking place before and after the switch from a mitotic cycle to an endo-replication cycle. It can be anticipated that the major pool of somatic piRNAs is produced during the first period when piRNA precursors exit from the nucleus and Yb-bodies are correctly assembled.

Overall, these data show that in addition to factors specifically required for piRNA biogenesis, the spatio-temporal regulation of the whole system takes place at another level of complexity, the understanding of which will certainly help to interpret delayed and unexpected regulations.

## Methods

### Drosophila stocks

The used fly strains were: *ISO1A* from the collection of the GReD; *armi* [1]/*TM3* and *armi* [72.1]/*TM6*and RNAi line *nxf1* (34945) from Bloomington *Drosophila* Stock Center; RNAi lines: *armi* (103589KK) and *yb* (110056KK) from Vienna Drosophila RNAi Centre.

### Cell culture

OSS cells were grown in prepared from Shields and Sang M3 Insect Medium (Sigma) supplemented with 0.6 mg/ml glutathione, 10 mU/ml insulin, 10% fetal bovine serum and 10% fly extract.

### RNA fish

The DNA fragment to prepare the specific *flam* 508 probe to detect *flam* transcripts was PCR amplified from the *ISO1A* line using primers 5′-ATTCTCCTTTCTCAGGATGC-3′ and 5′-GCATTGCTACCTTACGTTTC-3′ and cloned into pGEMT easy vector.

Riboprobe was synthesized by digestion of pGEMT easy plasmids with NcoI or SpeI enzyme, followed by in vitro transcription using Sp6 or T7 polymerase and digoxygenin labeled UTP (Roche), DNAse I treatment and purification.

RNA FISH on ovaries was performed as previously described [13]. In situ hybridization on OSS cells was carried out essentially described for ovaries. OSS cells were fixed in 4% formaldehyde/PBT (1X PBS, 0.1% Triton) at RT for 30 min, rinsed three times with PBT and post-fixed 10 min in 4% formaldehyde/PBT. After washes in PBT and permeabilisation (1 h in 1X PBS,0.3% Triton) prehybridization was done as follow: 10 min HYB- (50% Formamide, 5X SSC, 0.02% Tween)/PBT 1:1, 10 min HYB-, 1 h HYB+ (HYB- with yeast tRNA 0.5 μg/μl, 0.25 mg/ml heparin) at 37 °C. Ovaries were hybridized overnight at 37 °C with 1 μg riboprobe previously denaturated 10 min at 74 °C. Ovaries were then rinsed 20 min in HYB- and in HYB−/PBT at 37 °C then 4 times in PBT at RT before blocking 1 h at RT in TNB (Perkin-Elmer TSA kit) and immunodetection 1 h30 at RT with anti-Dig-HRP (Roche) in TNB 0.3% Triton. Cells were rinsed three times in PBT, incubated 10 min with TSA-Cy5 in amplification diluent (Perkin-Elmer) and rinsed three times in PBT.

When coupled to immunofluorescence, RNA straining was followed by incubation with mouse anti-lamin antibody (ADL67–10, Hybridoma), goat anti-Armitage antibody (sc-34,564, Santa Cruz), Yb antibody (kindly provided by G. Hannon), GAPDH antibody (IMG-5143A-050, Imgenex) Secondary antibodies coupled to Cy3 or Alexa-488 were used.

### Immunofluorescence

Ovaries from 2-to 4-days-old flies were dissected in PBT fixed in 4% formaldehyde/PBT at RT for 20 min, rinsed three times with PBT, incubated 1 h in PBS-0.3% Triton, rinsed three times with PBT and incubated 1 h in TNB 0,3% Triton prior staining with anti-lamin, anti-Armitage or anti- Yb antibody. Secondary antibodies coupled to Cy3, Cy-5 or Alexa-488 were used.

Three-dimensional images were acquired on Leica SP5 and Leica SP8 confocal microscopes using a 40X objective, acquiring a stack of at least 10 slices with an interval of 0,5 nm for each follicle to ensure signal detection within the entire volume of the follicle cells. Only cells acquired in their totality were considered for quantifications. Manual scoring was performed for the presence/absence of *flam* RNA signal in follicle cells (Fig. 1a) and for the localization of *flam* RNA probe signal compared to lamin signal (Figs. 1b, 2b and 4b). The presence/absence of Armi and Yb foci in each follicle cell was assessed manually (Fig. 3b).

## Additional files

Additional file 1: **Figure S1.** Pattern of Armi and Yb staining in OSS cells and in whole ovariole (A) *flam* transcripts (red), nuclear membrane (green) and Armi (white) (top) or Yb-foci (white) (bottom) are visualized by RNA-FISH and immunofluorescence using *flam* 508 RNA probe, anti-lamin antibody, anti-Armi and anti-Yb antibodies in OSS cells. (B) Armi (red) and Yb-foci (white) and nuclear membrane (green) are visualized in whole ovarioles by immunofluorescence using respectively anti-Armi, anti-Yb and anti-lamin antibodies. A clear drop in Armi and Yb staining is visualized in the surrounding follicle cells, in early versus late stages of oogenesis (TIF 142 kb)

Additional file 2: **Figure S2.** Unusual nuclear localization of Armi in follicle cells of late stages in *Nxt1*-SKD flies Armi but not Yb protein can occasionally be visualized within the nucleus of follicle cells of late stages of oogenesis in *Nxt1*-SKD flies. Armi (red), and Yb protein (white) and nuclear membrane (green) are visualized by immunofluorescence using anti-Armi, anti-Yb, and anti-lamin antibody in follicle cells of *Nxt1*-SKD egg chambers of stage 10 (TIF 1479 kb)

Additional file 3: **Table S1.** Number of cells and follicles analyzed for quantifications presented in Figs. 1a-b, 2b, 3b, 4b (XLSX 11 kb)

## Data Availability

All data generated or analysed during this study are included in this published article [and its supplementary information files].
