# Supplementary figures and images for: flam piRNA precursors channel from the nucleus to the cytoplasm in a temporally regulated manner along Drosophila oogenesis
Source: Mob DNA. 2019 Jul 6;10:28. doi: 10.1186/s13100-019-0170-7 (PMC6612187; doi:10.1186/s13100-019-0170-7)

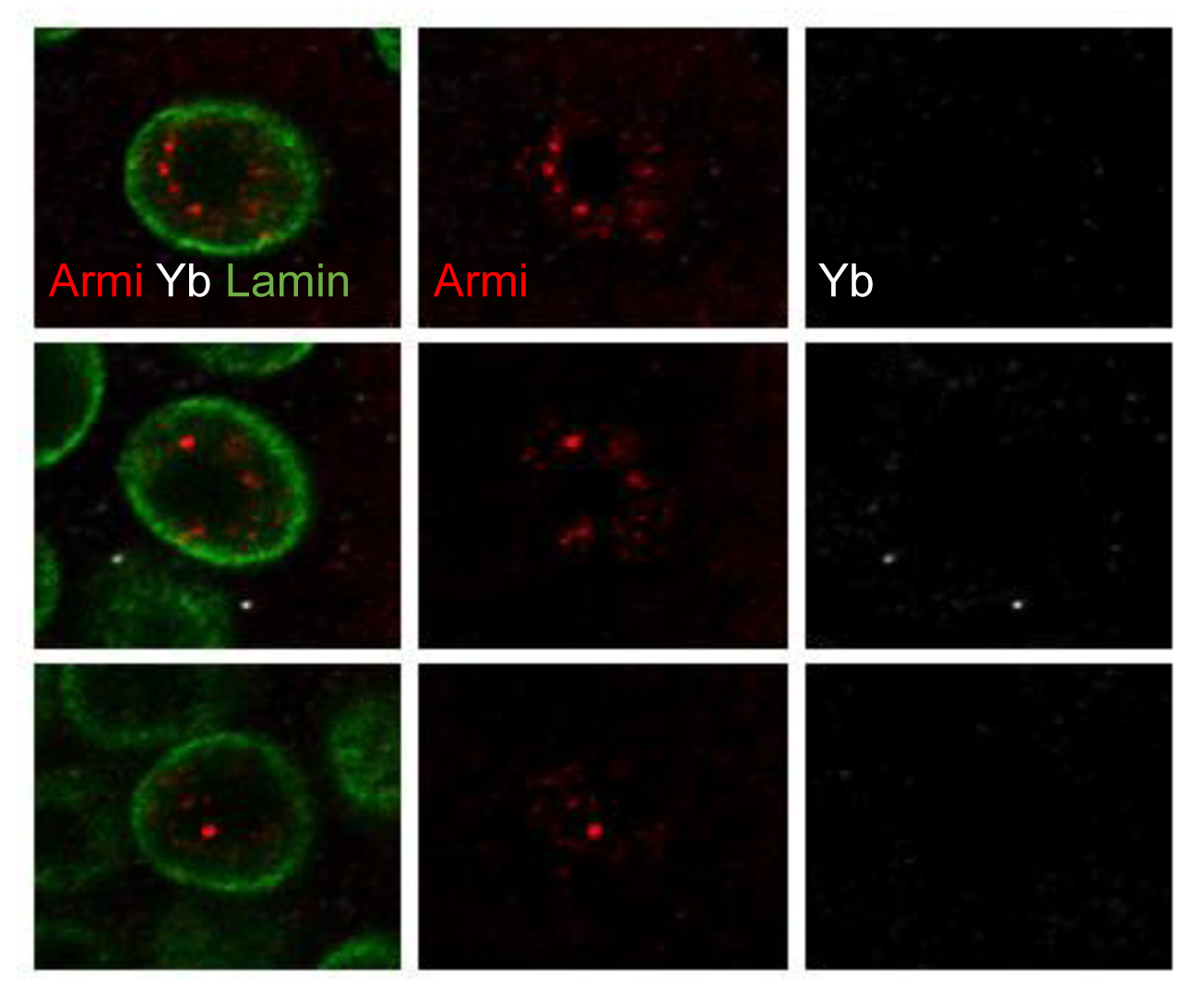

Supplement: Supplementary file 2 — Figure S2. Unusual nuclear localization of Armi in follicle cells of late stages in Nxt1-SKD flies Armi but not Yb protein can occasionally be visualized within the nucleus of follicle cells of late stages of oogenesis in Nxt1-SKD flies. Armi (red), and Yb protein (white) and nuclear membrane (green) are visualized by immunofluorescence using anti-Armi, anti-Yb, and anti-lamin antibody in follicle cells of Nxt1-SKD egg chambers of stage 10 (TIF 1479 kb) [file 13100_2019_170_MOESM2_ESM.tif]
